# 2-[(3-Bromo­phen­yl)imino­meth­yl]phenol

**DOI:** 10.1107/S1600536808027360

**Published:** 2008-08-30

**Authors:** Xiao-Yan Ren, Fang-Fang Jian

**Affiliations:** a Microscale Science Institute, Weifang University, Weifang 261061, People’s Republic of China

## Abstract

The title compound, C_13_H_10_BrNO, was prepared by reaction of 3-bromo­aniline with 2-hydroxy­benzaldehyde at 377 K. The mol­ecular structure and packing are stabilized by an intra­molecular O—H⋯N hydrogen-bond inter­action.

## Related literature

For related literature, see: Jian *et al.* (2006 ▶); Rozwadowski *et al.* (1999 ▶).
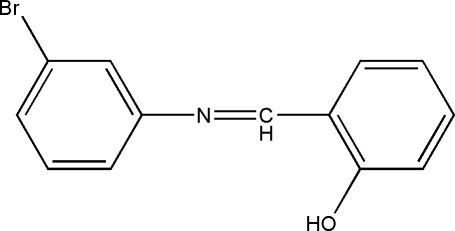


## Experimental

### 

#### Crystal data

C_13_H_10_BrNO
*M* *_r_* = 276.13
Monoclinic, 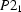

*a* = 3.9700 (8) Å
*b* = 10.540 (2) Å
*c* = 13.200 (3) Å
β = 98.00 (3)°
*V* = 546.96 (19) Å^3^
*Z* = 2
Mo *K*α radiation
μ = 3.73 mm^−1^
*T* = 293 (2) K
0.12 × 0.10 × 0.07 mm

#### Data collection

Bruker SMART CCD area-detector diffractometer
Absorption correction: none
2736 measured reflections
1822 independent reflections
1666 reflections with *I* > 2σ(*I*)
*R*_int_ = 0.032

#### Refinement

*R*[*F* ^2^ > 2σ(*F* ^2^)] = 0.082
*wR*(*F* ^2^) = 0.229
*S* = 1.13
1822 reflections
145 parameters
1 restraint
H-atom parameters constrained
Δρ_max_ = 1.43 e Å^−3^
Δρ_min_ = −1.17 e Å^−3^
Absolute structure: Flack (1983 ▶), 787 Freidel pairs
Flack parameter: 0.1 (4)

### 

Data collection: *SMART* (Bruker, 1997 ▶); cell refinement: *SAINT* (Bruker, 1997 ▶); data reduction: *SAINT*; program(s) used to solve structure: *SHELXS97* (Sheldrick, 2008 ▶); program(s) used to refine structure: *SHELXL97* (Sheldrick, 2008 ▶); molecular graphics: *SHELXTL* (Sheldrick, 2008 ▶); software used to prepare material for publication: *SHELXTL*.

## Supplementary Material

Crystal structure: contains datablocks global, I. DOI: 10.1107/S1600536808027360/at2611sup1.cif

Structure factors: contains datablocks I. DOI: 10.1107/S1600536808027360/at2611Isup2.hkl

Additional supplementary materials:  crystallographic information; 3D view; checkCIF report

## Figures and Tables

**Table 1:** Hydrogen-bond geometry (Å, °)

*D*—H⋯*A*	*D*—H	H⋯*A*	*D*⋯*A*	*D*—H⋯*A*
O1—H1⋯N1	0.82	1.86	2.599 (17)	149

**Fig. 1.**
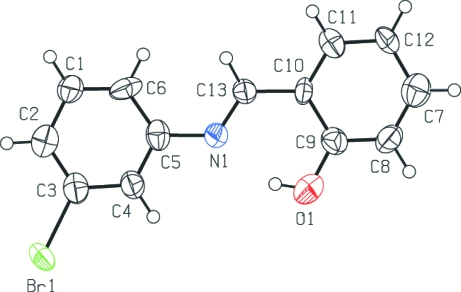
The molecular structure of the title compound with the atom-labeling scheme. Displacement ellipsoids are drawn at the 50% probability level.

**Fig. 2.**
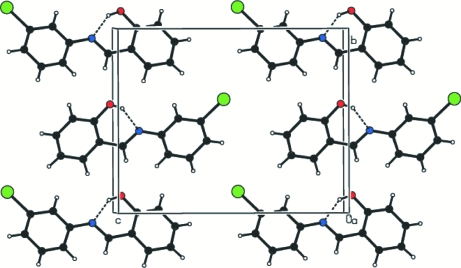
A view of the packing and the intramolecular hydrogen bonding (dashed lines) of (I) in the unitcell.

## supplementary crystallographic information

### Comment

The recent growing interest in Schiff bases is also due to their ability to form intramolecular hydrogen bonds by electron coupling between acid–base centers (Rozwadowski *et al.*, 1999). The part of our research is to find Schiff base with higher biological activity, we synthesized the title compound (I) and report its crystal structure here.

In the crystal structure of compound (I) (Fig. 1), the dihedral angle between the benzene rings (C1–C6) and (C7–C12) was 4.6 (2)°. The C═N bond length [1.273 (1) Å] is in agreement with that observed before (Jian *et al.*, 2006). There are intramolecular O—H···N hydrogen-bond interactions to stabilize the crystal structure (Table 1, Fig. 2).

### Experimental

A mixture of 2-nitrobenzaldehyde (0.02 mol) and 4-methoxyaniline (0.02 mol) was stirred with ethanol (50 mL) at 377 K for 5 h, affording the title compound (4.33 g, yield 84.5%). Single crystals suitable for X-ray measurements were obtained by recrystallization from acetone at room temperature.

### Refinement

H atoms were positioned geometrically and allowed to ride on their parent atoms, with O—H and C—H distances of 0.82 and 0.93 Å, respectively, and with *U*_iso_(H) = 1.2 or 1.5*U*_eq_ of the parent atoms.

### Crystal data

**Table:**

C_13_H_10_BrNO	*F*_000_ = 276.0
*M**_r_* = 276.13	*D*_x_ = 1.676 Mg m^−^^3^
Monoclinic, *P*2_1_	Mo *K*α radiation λ = 0.71073 Å
Hall symbol: P 2yb	Cell parameters from 1666 reflections
*a* = 3.9700 (8) Å	θ = 1.6–25.0º
*b* = 10.540 (2) Å	µ = 3.73 mm^−^^1^
*c* = 13.200 (3) Å	*T* = 293 (2) K
β = 98.00 (3)º	Bar, yellow
*V* = 546.96 (19) Å^3^	0.12 × 0.10 × 0.07 mm
*Z* = 2	

### Data collection

**Table:**

Bruker SMART CCD area-detector diffractometer	1666 reflections with *I* > 2σ(*I*)
Radiation source: fine-focus sealed tube	*R*_int_ = 0.032
Monochromator: graphite	θ_max_ = 25.0º
*T* = 293(2) K	θ_min_ = 1.6º
φ and ω scans	*h* = −4→4
Absorption correction: none	*k* = −12→12
2736 measured reflections	*l* = −12→15
1822 independent reflections	

### Refinement

**Table:**

Refinement on *F*^2^	Hydrogen site location: inferred from neighbouring sites
Least-squares matrix: full	H-atom parameters constrained
*R*[*F*^2^ > 2σ(*F*^2^)] = 0.082	*w* = 1/[σ^2^(*F*_o_^2^) + (0.1154*P*)^2^ + 2.8393*P*] where *P* = (*F*_o_^2^ + 2*F*_c_^2^)/3
*wR*(*F*^2^) = 0.229	(Δ/σ)_max_ < 0.001
*S* = 1.13	Δρ_max_ = 1.43 e Å^−^^3^
1822 reflections	Δρ_min_ = −1.17 e Å^−^^3^
145 parameters	Extinction correction: none
1 restraint	Absolute structure: Flack (1983), 787 Freidel pairs
Primary atom site location: structure-invariant direct methods	Flack parameter: 0.1 (4)
Secondary atom site location: difference Fourier map	

### Special details

**Table:**

Geometry. All e.s.d.'s (except the e.s.d. in the dihedral angle between two l.s. planes) are estimated using the full covariance matrix. The cell e.s.d.'s are taken into account individually in the estimation of e.s.d.'s in distances, angles and torsion angles; correlations between e.s.d.'s in cell parameters are only used when they are defined by crystal symmetry. An approximate (isotropic) treatment of cell e.s.d.'s is used for estimating e.s.d.'s involving l.s. planes.
Refinement. Refinement of *F*^2^ against ALL reflections. The weighted *R*-factor *wR* and goodness of fit *S* are based on *F*^2^, conventional *R*-factors *R* are based on *F*, with *F* set to zero for negative *F*^2^. The threshold expression of *F*^2^ > σ(*F*^2^) is used only for calculating *R*-factors(gt) *etc*. and is not relevant to the choice of reflections for refinement. *R*-factors based on *F*^2^ are statistically about twice as large as those based on *F*, and *R*- factors based on ALL data will be even larger.

### Fractional atomic coordinates and isotropic or equivalent isotropic displacement parameters (Å^2^)

**Table:**

	*x*	*y*	*z*	*U*_iso_*/*U*_eq_	
Br1	0.4943 (3)	0.6144 (2)	0.53030 (8)	0.0544 (5)	
N1	0.643 (3)	0.4449 (9)	0.9014 (8)	0.041 (2)	
C4	0.538 (4)	0.5109 (12)	0.7289 (10)	0.046 (3)	
H4A	0.6652	0.5830	0.7485	0.055*	
C10	0.793 (3)	0.3864 (10)	1.0766 (9)	0.036 (3)	
C3	0.402 (4)	0.4950 (13)	0.6277 (11)	0.049 (3)	
C11	0.800 (4)	0.2937 (12)	1.1480 (10)	0.043 (3)	
H11A	0.7186	0.2137	1.1274	0.052*	
C5	0.487 (3)	0.4229 (12)	0.8000 (10)	0.043 (3)	
C8	1.128 (4)	0.5145 (14)	1.2004 (12)	0.057 (4)	
H8A	1.2470	0.5889	1.2187	0.068*	
C12	0.918 (4)	0.3124 (13)	1.2476 (10)	0.052 (4)	
H12A	0.8763	0.2550	1.2978	0.062*	
C13	0.644 (4)	0.3630 (11)	0.9723 (11)	0.041 (3)	
H13A	0.5431	0.2846	0.9561	0.050*	
C9	0.978 (4)	0.4994 (12)	1.1041 (10)	0.045 (3)	
C7	1.110 (4)	0.4238 (15)	1.2716 (13)	0.060 (4)	
H7A	1.2254	0.4348	1.3373	0.072*	
O1	0.995 (3)	0.5906 (16)	1.0355 (8)	0.081 (6)	
H1	0.8925	0.5681	0.9801	0.121*	
C6	0.304 (3)	0.3157 (12)	0.7675 (13)	0.050 (4)	
H6A	0.2798	0.2530	0.8157	0.060*	
C2	0.216 (4)	0.3883 (13)	0.5982 (12)	0.049 (3)	
H2A	0.1275	0.3769	0.5298	0.059*	
C1	0.157 (5)	0.2960 (13)	0.6708 (11)	0.054 (4)	
H1B	0.0231	0.2250	0.6527	0.065*	

### Atomic displacement parameters (Å^2^)

**Table:**

	*U*^11^	*U*^22^	*U*^33^	*U*^12^	*U*^13^	*U*^23^
Br1	0.0649 (8)	0.0635 (8)	0.0338 (6)	−0.0095 (9)	0.0028 (5)	0.0168 (7)
N1	0.053 (6)	0.034 (5)	0.034 (5)	−0.002 (4)	0.001 (5)	−0.001 (4)
C4	0.060 (8)	0.041 (7)	0.035 (7)	0.005 (6)	−0.002 (6)	0.001 (5)
C10	0.042 (7)	0.035 (6)	0.026 (6)	0.004 (5)	−0.009 (5)	−0.007 (5)
C3	0.053 (8)	0.053 (8)	0.040 (7)	0.006 (6)	0.000 (6)	−0.002 (6)
C11	0.052 (8)	0.045 (7)	0.035 (7)	0.015 (6)	0.009 (6)	0.002 (6)
C5	0.039 (7)	0.049 (7)	0.038 (7)	0.013 (6)	−0.002 (5)	−0.002 (5)
C8	0.061 (9)	0.043 (7)	0.060 (9)	−0.009 (6)	−0.015 (7)	−0.003 (6)
C12	0.073 (10)	0.045 (7)	0.034 (7)	0.001 (7)	−0.007 (6)	0.013 (6)
C13	0.060 (8)	0.028 (6)	0.034 (7)	0.004 (5)	0.000 (6)	0.000 (4)
C9	0.056 (8)	0.039 (6)	0.041 (7)	0.007 (6)	0.003 (6)	0.003 (6)
C7	0.052 (9)	0.066 (9)	0.060 (9)	0.002 (7)	−0.001 (7)	−0.005 (7)
O1	0.096 (8)	0.077 (16)	0.065 (7)	−0.043 (8)	−0.005 (6)	−0.004 (7)
C6	0.035 (7)	0.032 (6)	0.079 (10)	−0.009 (5)	−0.002 (7)	0.004 (6)
C2	0.039 (7)	0.059 (9)	0.049 (8)	0.003 (6)	0.006 (6)	−0.010 (7)
C1	0.084 (11)	0.043 (8)	0.037 (7)	−0.011 (7)	0.014 (7)	−0.013 (6)

### Geometric parameters (Å, °)

**Table:**

Br1—C3	1.872 (15)	C8—C7	1.35 (2)
N1—C13	1.273 (17)	C8—H8A	0.9300
N1—C5	1.414 (16)	C12—C7	1.41 (2)
C4—C5	1.354 (19)	C12—H12A	0.9300
C4—C3	1.379 (19)	C13—H13A	0.9300
C4—H4A	0.9300	C9—O1	1.328 (19)
C10—C11	1.355 (18)	C7—H7A	0.9300
C10—C9	1.420 (18)	O1—H1	0.8200
C10—C13	1.443 (18)	C6—C1	1.34 (2)
C3—C2	1.37 (2)	C6—H6A	0.9300
C11—C12	1.348 (19)	C2—C1	1.41 (2)
C11—H11A	0.9300	C2—H2A	0.9300
C5—C6	1.381 (18)	C1—H1B	0.9300
C8—C9	1.34 (2)		
			
C13—N1—C5	122.7 (11)	C7—C12—H12A	121.8
C5—C4—C3	120.8 (13)	N1—C13—C10	123.0 (11)
C5—C4—H4A	119.6	N1—C13—H13A	118.5
C3—C4—H4A	119.6	C10—C13—H13A	118.5
C11—C10—C9	117.9 (11)	O1—C9—C8	120.3 (13)
C11—C10—C13	120.4 (11)	O1—C9—C10	120.4 (11)
C9—C10—C13	121.1 (11)	C8—C9—C10	119.3 (12)
C2—C3—C4	119.8 (14)	C8—C7—C12	120.8 (14)
C2—C3—Br1	120.4 (11)	C8—C7—H7A	119.6
C4—C3—Br1	119.7 (11)	C12—C7—H7A	119.6
C12—C11—C10	122.9 (13)	C9—O1—H1	109.5
C12—C11—H11A	118.6	C1—C6—C5	124.1 (13)
C10—C11—H11A	118.6	C1—C6—H6A	117.9
C4—C5—C6	117.9 (13)	C5—C6—H6A	117.9
C4—C5—N1	117.2 (12)	C3—C2—C1	120.5 (14)
C6—C5—N1	124.8 (13)	C3—C2—H2A	119.8
C9—C8—C7	121.2 (14)	C1—C2—H2A	119.8
C9—C8—H8A	119.4	C6—C1—C2	116.7 (13)
C7—C8—H8A	119.4	C6—C1—H1B	121.6
C11—C12—C7	116.5 (14)	C2—C1—H1B	121.6
C11—C12—H12A	121.8		

### Hydrogen-bond geometry (Å, °)

**Table:**

*D*—H···*A*	*D*—H	H···*A*	*D*···*A*	*D*—H···*A*
O1—H1···N1	0.82	1.86	2.599 (17)	149
